# The somatic mutation landscape of Chinese Colorectal Cancer

**DOI:** 10.7150/jca.37017

**Published:** 2020-01-01

**Authors:** Rong Ma, Changwen Jing, Yuan Zhang, Haixia Cao, Siwen Liu, Zhuo Wang, Dan Chen, Junying Zhang, Yang Wu, Jianzhong Wu, Jifeng Feng

**Affiliations:** 1 Clinical Cancer Research Center, Jiangsu Cancer Hospital &Jiangsu Institute of Cancer Research &The Affiliated Cancer Hospital of Nanjing Medical University, China.; 2 Department of Chemotherapy, Jiangsu Cancer Hospital &Jiangsu Institute of Cancer Research &The Affiliated Cancer Hospital of Nanjing Medical University, China.

**Keywords:** Colorectal cancer, Whole exome-sequencing, FCGBP, NBPF1

## Abstract

Colorectal cancer (CRC) is the fifth leading cause of cancer-related death in China. The incidence of Chinese CRC has increased dramatically with the changes of dietary and lifestyle. However, the genetic landscape of Chinese colorectal cancer mutation is still poorly understood. In this study, we have performed whole exome-sequencing analysis of 63 CRC cases. We found that Chinese CRC were hypermutated, which were enriched in ECM-receptor interaction, antigen processing and presentation, and focal adhesion. Analysis with clinical characteristics indicated that the deficiency of CRC driver gene, FCGBP and NBPF1 conferred CRC development and was showed worse survival rates, which could be the novel regulators and, diagnostic and prognostic biomarkers for Chinese CRC. Taken together, the application of whole exome-sequencing unveiled previously unsuspected somatic mutation landscape in Chinese CRCs, which may expand the understanding of disease mechanisms and provide an alternative personalized treatment for Chinese CRC patients.

## Introduction

Colorectal cancer (CRC) is one of the most common malignant diseases worldwide, which ranks third (10.2%) in terms of incidence but second (9.2%) in terms of mortality^1^. With more than 1.8 million new cases and 881,000 deaths each year, which are estimated to occur in 2018^1^. Meanwhile, the incidence and mortality of CRC are constantly increasing, and CRC is the fifth leading cause of death in China^2^. Over 50% of CRC patients develop colorectal metastasis involved the liver, lungs and lymph nodes with high mortality 3.

CRC develops through a series of germline or somatic mutations, which affect the homeostasis of oncogenes or tumor suppressors. With the advances in whole exome sequencing (WES) and whole genome sequencing (WGS) combining the other multi-omics studies, a large proportion of somatic mutations in CRC were identified, including *TP53*, *APC*, *TTN*, *KRAS*, *PIK3CA*, *SMAD4*, *FBXW7* and *RNF43*, which drive the evolution of a malignant CRC^4^. Putative driver mutations in 29 genes associated with, including the mismatch repair and pathways. DNA damage response and repair (DRR) disorder (DNA mismatch repair and homologous recombination, Chromosomal instability (CIN), and the CpG island methylation phenotype (CIMP), have been implicated in CRC development. Mutations in DNA mismatch repair genes (MLH1, MLH3, MSH2, MSH3, MSH6, and PMS2) or hypermethylation of MLH1 typically leads to microsatellite instability (MSI)^5^. Genetic defects in homologous recombination pathways were associated with genomic instability in microsatellite stability (MSS) CRC^5^.

Whole exome sequencing studies on American-African, Middle Eastern, Iranian and Japanese CRC patients uncovered significantly different somatic mutations, which indicated alternative CRC development with different regions and ethnic background 6-9. Although the CRC incidence and mortality rates in China are lower than in parts of Europe, Australia, Northern America, and Eastern Asia (Japan and the Republic of Korea), but which tends to increase rapidly in the most recent decade^1^. Therefore, it is necessary to characterize the somatic mutation landscape of Chinese CRC patients, and find the novel CRC risk factors, which could be established as predictive and prognostic markers in CRC. Several studies of Chinese CRC using WES have reported some novel somatic mutation genes, such as *CDH10* (8.2%), *FAT4* (14.3%), *DOCK2* (7.7%), *PCDHB3* (5.19%), *PEG3* (10.6%) and *TMEM128* (4/10) 10-13, but mutation frequency characterization is limited in the sample size, sequencing depth and coverage. However, the genome-wide somatic mutations involved in CRC progression are poorly understood in Chinese patients.

To identify the catalog of CRC risk loci and improve our understanding of somatic mutations influencing Chinese CRC development, we performed WES analysis of 63 Chinese CRC cases. In this study, we found that Chinese CRC were hypermutated at a high depth of average coverage (~185X). Novel somatic mutations were enriched in ECM-receptor interaction, antigen processing and presentation, and focal adhesion. Our clinical analysis revealed FCGBP and NBPF1 could be the novel regulators and, diagnostic and prognostic biomarkers for Chinese CRC. These new somatic mutation genes would be the novel regulators and, diagnostic and prognostic biomarkers for Chinese CRC, and provide new potential therapy targets.

## Materials and Methods

### Patients

Human primary colorectal cancer tumors and corresponding adjacent non-tumor tissues (5 cm from the tumor site) were obtained from 63 Chinese patients, who were recruited in Jiangsu province, China. Clinical characteristics of all patients were listed with detailed information summarized in Supplementary Table S1. The histological diagnosis of all samples was confirmed by the pathologists. Tumor stage was determined by TNM classification of malignant tumors. All patients participated in the study signed informed consent. The ethics approval was awarded by Jiangsu Institute of Cancer Research Ethics Committee.

### Whole exome sequencing analysis

Genomic DNA was extracted with DNA FFPE tissue kit (Omega, Norcross, GA, USA) following the manufacturer's recommendations, and the concentrations were detected by Qubit® 2.0 fluorometer dsDNA HS assay kit (Thermo Fisher Scientific, Oregon, USA).

Genomic DNA samples were randomly fragmented into sizes distributed between 200 and 300 bp using TIANSeq DNA Fragmentation Module Kit (Tiangen, China). DNA fragments were end repaired, and an adenylate blocker was added at the 3' end. Adaptors with barcode sequences were then ligated to both ends of the fragments. PEG/NaCl SPRI Solution and the beads were then used to select DNA fragments of the targeted size. Then, 9 cycles of polymerase chain reaction (PCR) were performed, and the mixtures were purified. Whole-exome capture was performed using SeqCap EZ Exome Library kits (Roche NimbleGen). After the libraries were amplified, the capture probes were added and incubated for 16-20 h at 47℃. The hybridized mixtures were amplified with another 14 cycles of PCR. All validated libraries were sequenced on the Illumina HiSeq X Ten.

### Exome sequencing data analysis

Reads with adapter contamination, reads containing uncertain nucleotides more than 10 percentage, and paired reads when single reads have more than 50 percentage low-quality (<5) nucleotides are discarded. Paired-end clean reads are aligned to the human reference genome (GRCh37/hg19) by using BWA0.7.10 14. BAM files were manipulated using SAMtools 15 and Picard tools (*http://picard.sourceforge.net*). Variant calling was performed following GATK4-4.0.4.0 16. Quality metrics were evaluated with at least 110X of the average depth per sample and achieved 10X coverage rate more than 93.8%. Variants obtained from previous steps were compared based on the asvsnp150 and 1000 Genomes database, followed by annotation with ANNOVAR 17. Variants with >1% frequency in the population variant databases 1000 Genomes Project, and subsequently intergenic, intronic, and synonymous variants were filtered, except those located at canonical splice sites. Somatic single nucleotide variations (SNVs) and somatic insertion-deletion (InDel) were identified with matched adjacent non-cancerous samples by using Mutect2 18.

### Gene Ontology and clinical data analyses

The following publicly available databases were utilized for the bioinformatics analysis: Kyoto Encyclopedia of Genes and Genomes (http://www.kegg.jp/kegg/pa), The Cancer Genome Atlas (https://cance rgenome.nih.gov/), UALCAN (http://ualcan.path.uab.edu/index.html) 19. We conducted pathway enrichment analysis with ClusterProfiler 20. Pathway with *P*-value ≤ 0.05, was considered to be significantly enriched.

### Significantly mutated genes analysis

We analyzed mutations with significantly mutated genes (SMGs) test based on tumor samples using MutSigCV1.41 21. Significantly mutated genes landscape heatplot was presented.

### Mutation spectrum and mutation signature analysis

We performed mutation spectrum and signature analysis to explore the relationship within tumor samples in each same patient by maftools 22. We also conducted clustering analysis on mutation spectrum to observe the similarity and difference within tumor samples using nonnegative matrix factorization 23.

## Results

### Exome sequence analysis

**Figure 1:**
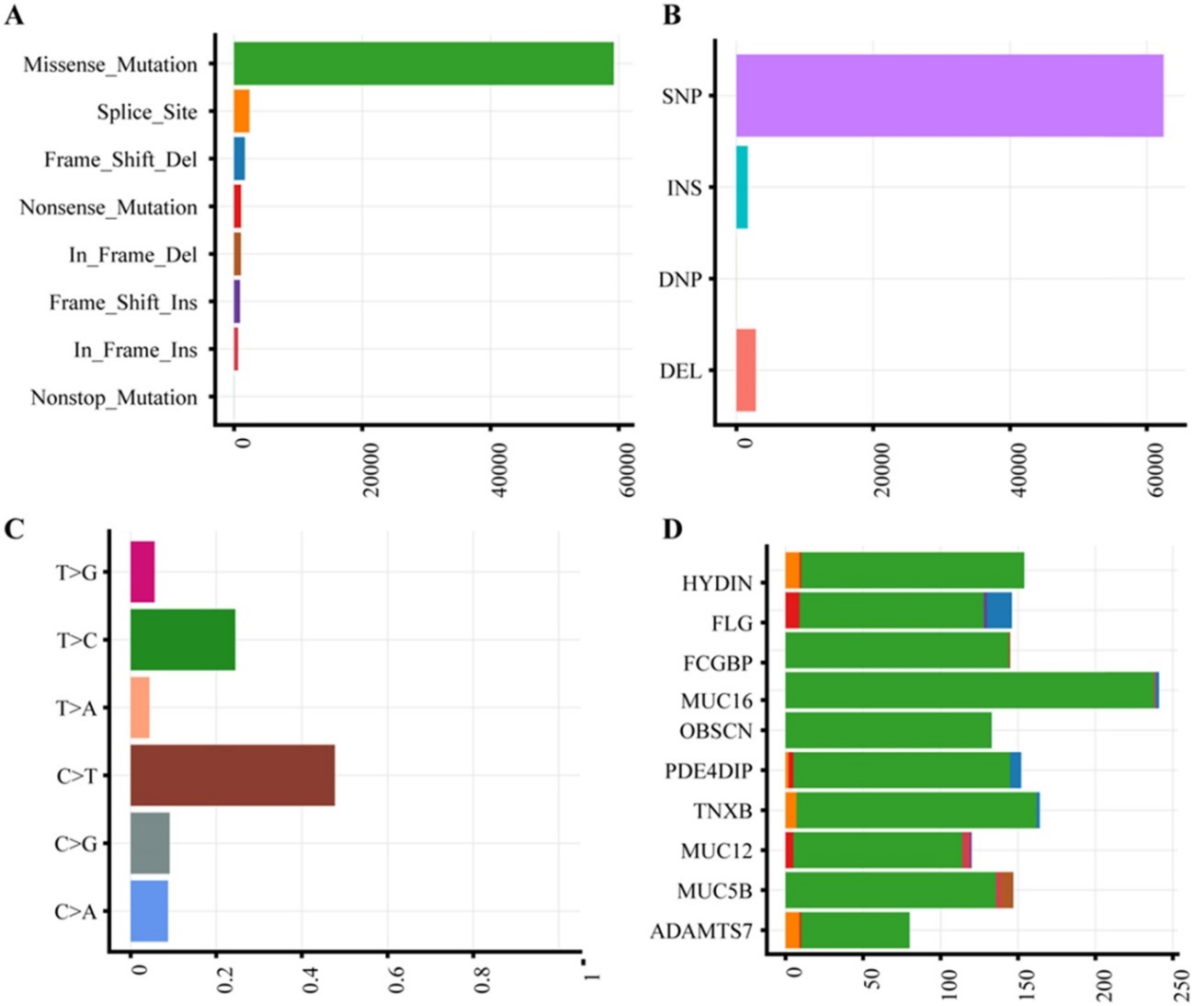
** Mutation plot summary of colorectal cancer samples. (A)** Variant classification. X axis indicated variant numbers. Y axis represented different variant classification. **(B)** Variant type. X axis indicated variant numbers. Y axis represented different variant type. **(C)** SNVs type. X axis indicated the ratio. Y axis represented the type of nucleotide substitution. **(D)** Top10 mutated genes. X axis indicated variant numbers. Y axis represented different genes. The genes were ordered by their mutation frequency.

The characteristics of 63 CRC patients were described in Supplementary Table 1. To identify somatic variants associated with CRC, we performed whole exome sequencing on the tumor and matched tissues. All samples were attained 185-fold average coverage and at least 93.8% mapping rate of targeted exons (Supplementary Fig. S1, Supplementary Table S2). In total, we detected 64,973 SNVs and 4519 InDels more than ever published data (Supplementary Table S3). Missense mutation and single nucleotide polymorphism are the most common variant type (Fig. 1A-B). The dominant enriched class of SNVs are C>T and T>C (Figure 1C).

### Mutation landscape of colorectal cancer

With the high depth of exome sequencing, we identified a different spectrum of somatic mutations in Chinese CRC compared to ever report. According to gene mutation frequency, the top 10 mutant genes were listed in Fig. 1D. *HYDIN* (88.89%) and *FLG* (88.89%) were top two mutation genes. Mutations in *HYDIN* caused hydrocephalus in mice 24, and impaired flagellar and ciliary motility 25, 26. Mice defective in *HYDIN* was early lethal within 3 weeks 25, 26. Filaggrin (FLG) is a key protein involved in epithelial barrier, and loss-of-function mutation of *FLG* caused ichthyosis vulgaris, Atopic dermatitis, skin microbiota and inflammation dysregulated diseases^27^. The expression of Fc fragment of the IgG binding protein (FCGBP, 87.30%) in mRNA and protein were both decreased in CRC 28, 29, which was reported that functioned as a regulator of TGF-1-induced epithelial-mesenchymal transition (EMT) 30. The three mucins, MUC16 (85.71%), MUC12 (73.02%) and MUC5B (71.43%), are the ideal biomarkers of CRC 31-33. The mutations of OBSCN (85.71%), PDE4DIP (82.54%), TNXB (73.02%) and ADAMTS7 (71.42%) were reported in previous study 34 and Genomic Data Commons (GDC) Data Portal. Chinese CRC somatic mutations showed the significantly different characteristics compared to the current public database, The Cancer Genome Atlas (TCGA) and Catalogue of Somatic Mutations in Cancer (COSMIC) (Supplementary Table S4). The Chinese CRC mutation frequency of TP53, APC, KRAS and PIK3CA was much less than western people, only TTN had a similar mutation rate.

**Figure 2:**
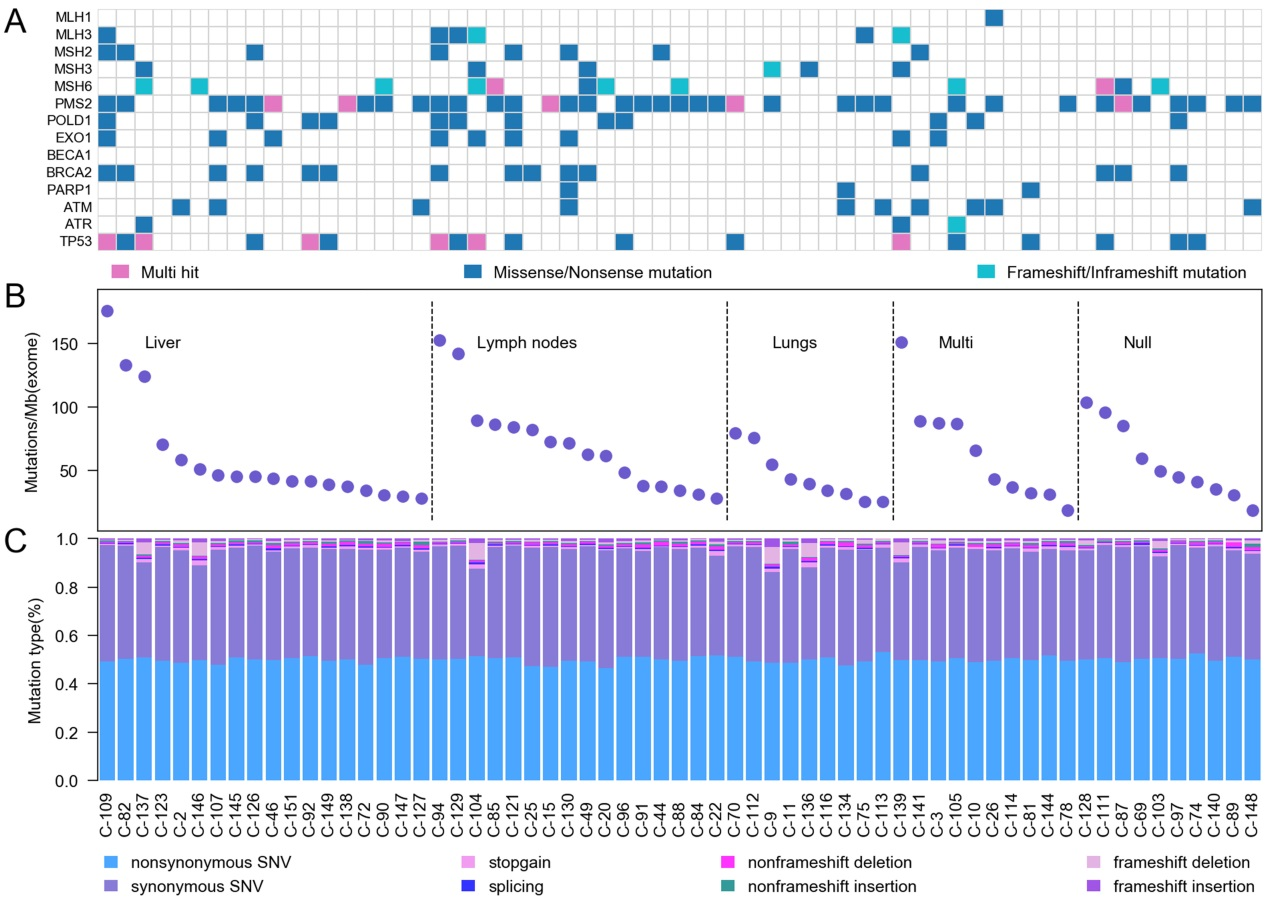
** Somatic mutation characteristics of Chinese CRC. A,** Mutation frequencies of DNA damage response and repair genes. **B,** Mutation frequency in 63 Chinese CRC. **C,** A display of the various categories of mutations across samples is shown for SNVs (non-synonymous SNV, synonymous SNV, stopgain SNV and splicing) and InDels (non-frameshift deletion, non-frameshift insertion, frameshift deletion and frameshift insertion).

We categorized our cases into three groups based on mutation rate combining with 14 DNA damage response and repair (DRR) genes^5^, including mismatch excision repair (MMR) genes and homologous recombination (HR) genes (Figure 2A). According to the location of tumor metastasis, we divided 63 CRC patients into five groups. 6 samples were ultra-hypermutated CRC, which the average nonsynonymous somatic mutations were 2513. 22 samples were defined as hypermutated CRC with average non-synonymous somatic mutation density of 1288 (Figure 2B). The hypermutated sample C-128 without upper 6 MMR genes displayed mutations on MSH4, which interrupted DNA binding and DNA replication. The other 35 CRC cases showed an average non-synonymous mutation density of 615. The mutation rates in our study were around 60/Mb for each sample (Figure 2C), which were much higher than previous Western and Chinese CRC 6-13. We found that PMS2 status was highly relevant to the highest mutation rates.

### Mutational signatures and disease ontology in Chinese CRC

**Figure 3:**
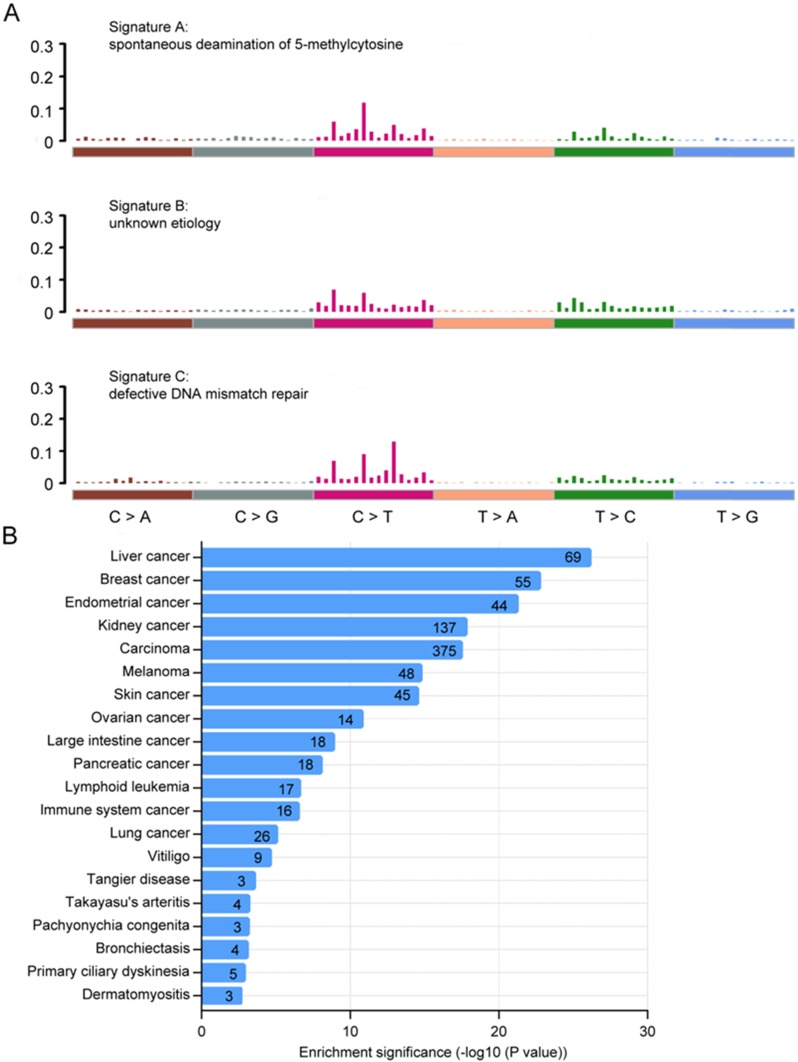
** Mutational signatures and disease ontology in Chinese CRC. A,** Three distinct mutation signatures (A-C) were extracted from Chinese CRCs and were shown according to cosine similarity, the correspondence is: Signature A and Signature 1 (age/spontaneous deamination of 5-methylcyotosine); Signature B and Signature 5 (unknown etiology; found in all cancer types); Signature C and Signature 6 (mismatch repair deficiency and microsatellite instability). **B,** Disease Ontology enrichment analysis for the somatic mutation genes in Chinese CRC.

We next analyzed the mutational signatures underlying the development of cancer in Chinese CRC. Three distinct signatures, designated A, B and C, were extracted from the mutation spectra of Chinese CRC (Fig. 3A), which were similar to signature 1, 5, 6 in COSMIC, with cosine similarity 0.804, 0.811 and 0.937, respectively. Signature A (COSMIC signature 1) was almost found in all cancer types, which was probably related to the spontaneous deamination of 5-methylcytosine and consistently correlated with age. Signature C (COSMIC signature 6) was most common in colorectal cancers, which was associated with defective DNA mismatch repair in 'microsatellite instability' cancers. Signatures A, B and C have previously been reported in sporadic colorectal cancers 35. Taken together, these results indicate that the mutational process in Chinese CRC was similar to sporadic colorectal cancer.

We also performed gene ontology (GO) analysis using the DAVID bioinformatics database of top 500 somatic mutation genes in Chinese CRC, and visualized GO terms with REVIGO 36, 37. We found that the somatic mutations in Chinese CRC might be involved in a variety of biological functions including cell adhesion, O-glycan processing, microtubule-based movement, immune response and cell differentiation (Supplementary Fig. S2A), which were significantly related to cancer. The most highly enriched pathways were ECM-receptor interaction, antigen processing and presentation, and focal adhesion (Supplementary Fig. S2B). What's more, we also performed an enrichment analysis based on Disease Ontology, and found that top 13 identified terms were all cancers including large intestine cancer.

### FCGBP is a potential regulator and novel biomarker for colorectal cancer

**Figure 4:**
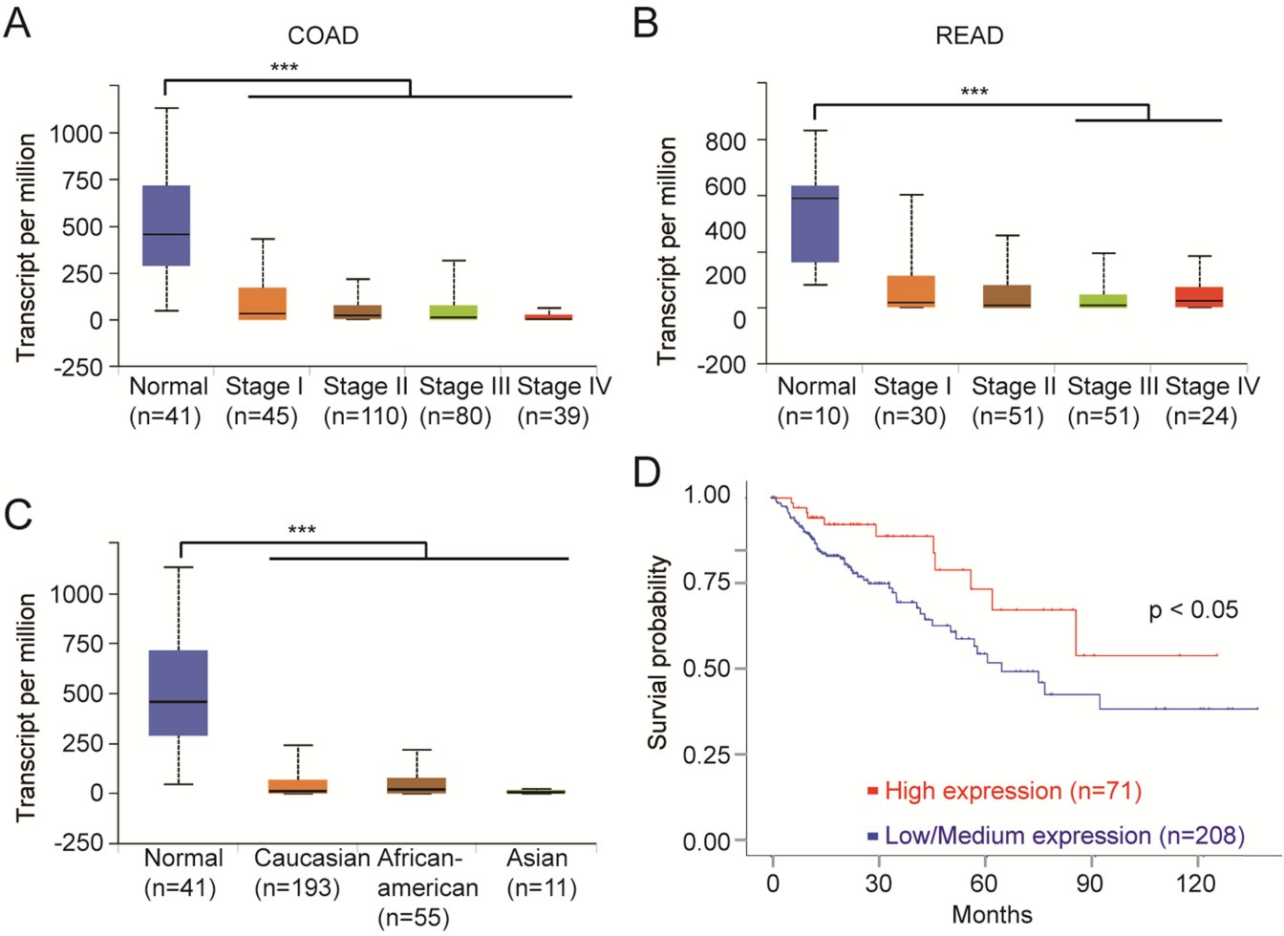
** FCGBP is a novel biomarker for CRC. A,** Expression levels of FCGBP in different stages of COAD from the TCGA database. **B,** Expression levels of FCGBP in different stages of READ from the TCGA database. **C,** Expression levels of FCGBP in normal, Caucasian, African American and Asian CRC patients. **D,** Lower expression levels of FCGBP indicate poor survival rate in CRC patients from the TCGA database.

We next analyzed the clinical data on the top 10 mutant genes using multiple bioinformatics tool sites from the TCGA database. The mRNA level of FCGBP was decreased in all stages of CRC tissues compared to normal tissues (Fig. 4A, B). And the relative expression of FCGBP in Caucasian, African American, and Asian CRC patients were much lower compared to normal cases (Fig. 4C). CRC with lower expression levels of FCGBP showed worse survival rates than cases with higher expression levels (Fig. 4D).

Notably, the expression of FCGBP protein was also decreased in CRC 28, 29. FCGBP might function as a mucin-like glycoprotein cooperated with MUC2, both of which were crucially essential part of mucus layers of the colon barrier 38, 39. FCGBP inhibited cancer cell migration and functioned as a regulator of TGF-1-induced epithelial-mesenchymal transition (EMT) 30. Together, FCGBP could be a potential regulator and novel biomarker for CRC.

### Identification of driver genes in Chinese CRC

**Figure 5:**
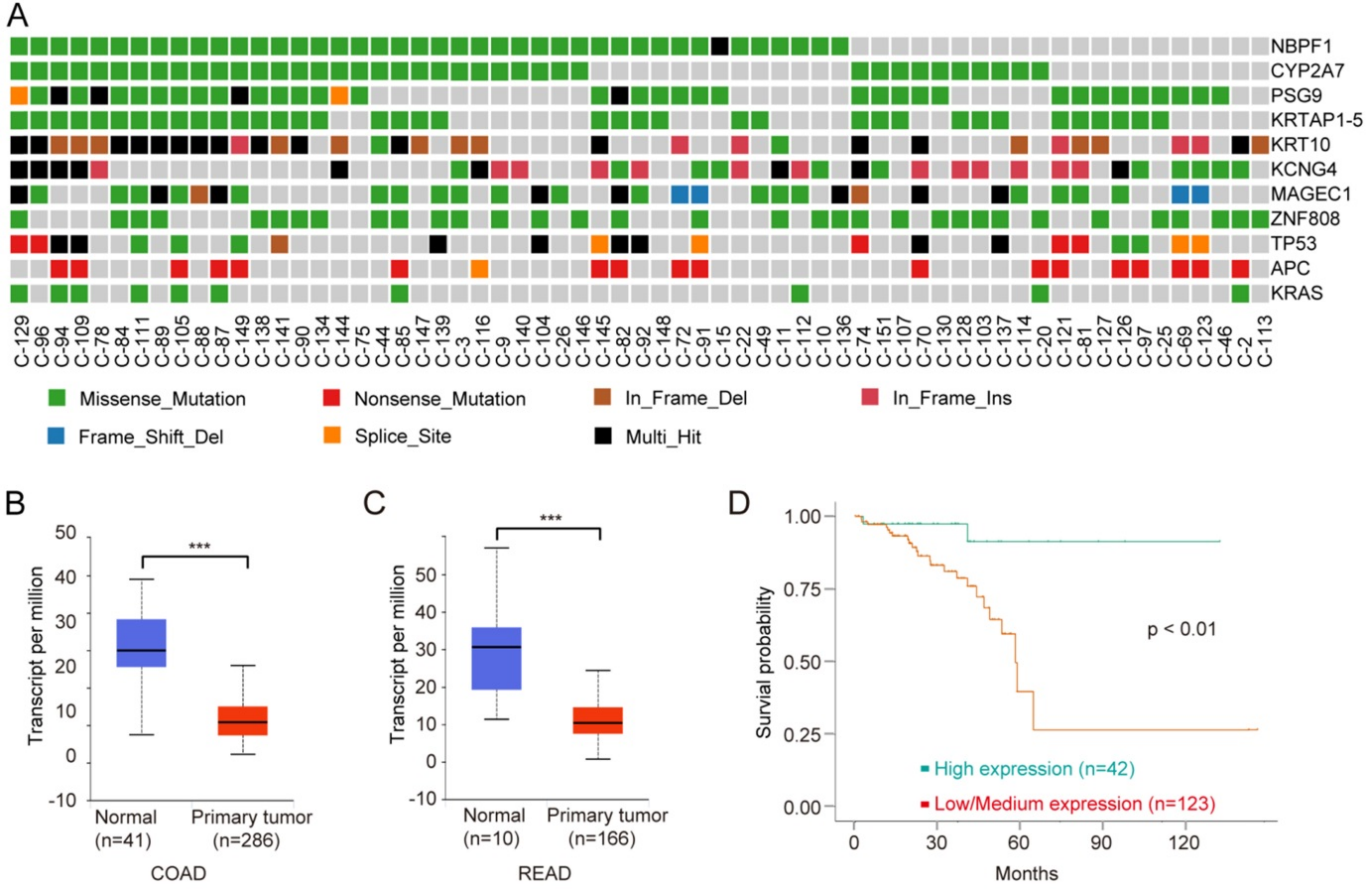
** NBPF1 is a SMG and novel biomarker for CRC. A,** Mutation frequencies of SMGs of Chinese CRC. **B,** Expression levels of NBPF1 in normal and COAD samples from the TCGA database. **C,** Expression levels of NBPF1 in normal and READ samples from the TCGA database. **D,** Lower expression levels of NBPF1 indicate poor survival rate in CRC patients from the TCGA database.

To identify driver genes in Chinese CRC, we used MutSigCV 21 to detect the susceptible significantly mutated gene (SMG). Overall, we identified 191 somatic recurrently mutated genes in Chinese CRC (q < 0.001, Supplementary Table S5), including two classical CRC genes, *TP53* and *APC*, which played key roles in CRC carcinogenesis. NBPF1 was the most frequently SMG in Chinese CRC (Fig. 5A). CYP2A7, PSG9, KRTAP1-5, KRT10, KCNG4, MAGEC1 and ZNF808 were also mutated frequently.

Neuroblastoma breakpoint Family member 1 (NBPF1) is a tumor suppressor, and the expression of NBPF1 is decreased in tumors 40. NBPF1 inhibits cell growth through inducing a G1 cell cycle arrest and control cell apoptosis via regulating PI3K-mTOR pathway 41, 42. We next analyzed the clinical data on NBPF1 using multiple bioinformatics tool sites from the TCGA database. The mRNA level of NBPF1 was decreased in CRC tissues compared to normal tissues (Fig. 5B, C). CRC with lower expression levels of NBPF1 showed worse survival rates than cases with higher expression levels (Fig. 5D). In conclusion, NBPF1, a tumor suppressor, could be a potential regulator and biomarker for CRC.

In addition, we used OncodriveFM (https://bitbucket.org/bbglab/oncodrivefm/src/master/) to analyze driver genes and combined the results with MutSigCV. The final results are listed in Table S6.

## Discussion

Whole exome sequencing of colorectal cancer unveils the specific driver mutation gene, which are associated with CRC development. Although earlier studies have shown a universal etiology for CRC 4, Chinese CRC patients have distinct landscape of somatic gene mutations and epidemiological features. Chinese CRC has a lower incidence rates than in Northern America, Eastern Asia (Japan and the Republic of Korea, Singapore), Australia, New Zealand, Europe, but higher compared with development transitioning countries in South Central Asia and Africa 1. Unfortunately, both incidence and mortality of Chinese CRC is increasing due to the dietary and lifestyle changes. However, the genetic characteristics of Chinese CRC were poorly understood, and urgent to be further studied. In this study, we investigated the somatic mutation landscape of Chinese CRC by whole exome sequencing.

The mutation characteristics of 63 Chinese CRC were hypermutated in this study, which were more likely shown to be MSI-like and genomic instability-like CRC. With the high depth of exome sequencing, we identified a different spectrum of somatic mutations in Chinese CRC compared to ever reported studies. We categorized 63 Chinese CRC cases into three groups based on mutation rates combining with MMR genes and HR genes. The mutation rates in our study were around 60/Mb for each sample, which were much higher than previous Western and Chinese CRC 6-13. According to gene mutation frequency, the top mutant genes were not APC or TP53, but *HYDIN*, *FLG*, FCGBP, MUC16, MUC12, MUC5B, OBSCN, PDE4DIP, TNXB and ADAMTS7. Chinese CRC somatic mutations showed the significantly different characteristics compared to the current public database TCGA and COSMIC. In this study, the mutation frequency of APC, KRAS and PIK3CA was much less than the populations of developed countries. To uncover the reason of this discrepancy, we will expand the Chinese CRC cases of WES to reduce the random errors.

GO analysis of top 500 somatic mutation genes in Chinese CRC indicates enrichment in cell adhesion, O-glycan processing, microtubule-based movement, immune response and cell differentiation, which were significantly associated to cancer. The most highly enriched pathways were ECM-receptor interaction, antigen processing and presentation, and focal adhesion. Disease Ontology was enriched in cancers including large intestine cancer. These results indicate that the specific genetic events are involved in the CRC development among the different populations.

FCGBP was first identified as an Fc fragment of the IgG binding protein in human small intestinal and colonic mucosa, which was secreted by these cells in human 39. FCGBP function as a mucin-like glycoprotein cooperated with MUC2, both of which were crucially essential part of mucus layers of the colon barrier, and might play a role in cell protection and inflammatory bowel disease 39. The expression of FCGBP in mRNA and protein were both decreased in CRC 28, 29, which was reported that functioned as a regulator of TGF-1-induced epithelial-mesenchymal transition (EMT) 30. The clinical data analysis indicates that the mRNA level of FCGBP was decreased in all stages of CRC tissues compared to normal tissues. CRC with lower expression levels of FCGBP showed worse survival rates than cases with higher expression levels. S853N and P278L on FCGBP gene were two novel hotspot mutations. Together, FCGBP could be a tumor suppressor and novel biomarker for CRC.

Additionally, SMG analysis identified a new CRC driver gene, NBPF1, which was not previously studied in CRC. NBPF1 is a member of the NBPF/DUF1220 domain family proteins, which was originally identified in a neuroblastoma (NB) patient with a constitutional translocation between chromosomes 1p36.2 and 17q11.2 40. The copy number of the NBPF/DUF1220 primarily located in chromosome 1, is much larger in humans than in other species, which NBPF/DUF1220 might play a vital role in human-specific evolution 43, 44. DLD1 cells, a colorectal cancer cell line, with increased NBPF1 expression had a decrease of clonal growth by a soft agar assay. The expression of NBPF1 is decreased in tumors, which functions as a tumor suppressor in neuroblastoma through inducing G1 cell cycle arrest 41. Moreover, NBPF1 regulates cell apoptosis via PI3K-mTOR pathway 42. And NBPF1 could be a DNA-binding transcription factor in nucleus, which was also a target of NF-κB 45. We also found that other members of NBPF/DUF1220 protein family, including NBPF4, NBPF11, NBPF12, NBPF14, NBPF16, NBPF20 and PDE4DIP, were also non-silently mutated. Although NBPF1 has been involved in several diseases, there is no report on the association of NBPF1 in CRC. The clinical data analysis indicates that the mRNA level of NBPF1 was decreased in CRC tissues compared to normal tissues. CRC with lower expression levels of NBPF1 showed worse survival rates than cases with higher expression levels. In conclusion, NBPF1, a tumor suppressor, could be also a potential regulator and biomarker for CRC.

Our study indicated a unique landscape of somatic mutations in Chinese CRC. The identification of new somatic mutations could provide new targets for personalized cancer treatment. We also report for the first time that two CRC driver gene, FCGBP and NBPF1 might function as tumor suppressors and prognostic markers for CRC. The roles of FCGBP and NBPF1 in the CRC development need further investigation.

## Supplementary Material

Supplementary figures and tables.
