# Supplementary material for: The somatic mutation landscape of Chinese Colorectal Cancer
Source: J Cancer. 2020 Jan 1;11(5):1038–46. doi: 10.7150/jca.37017 (PMC6959081; doi:10.7150/jca.37017)
Supplement: Supplementary file 1 — Supplementary figures and tables. [file jcav11p1038s1.pdf]

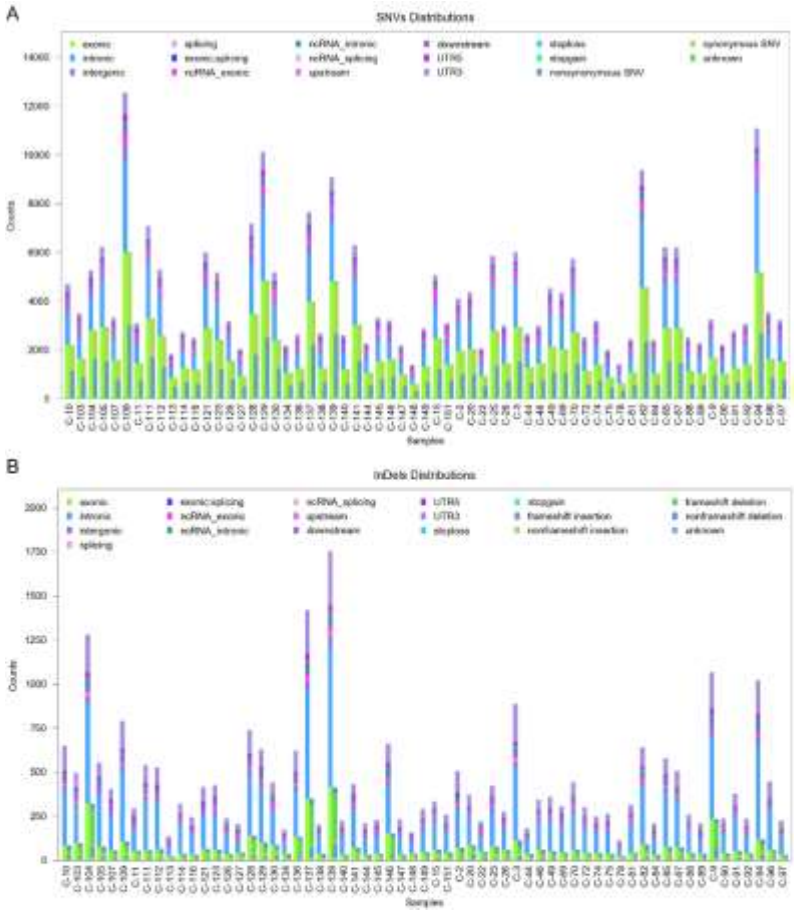

Supplementary Figure S1. Mutation distributions of SNVs and InDels in all colorectal cancer samples. A, SNVs distributions of all the samples. B, InDels distributions. The composition of SNV/InDels mutations was presented by different colors. X axis indicated different samples. Y axis represented the numbers.

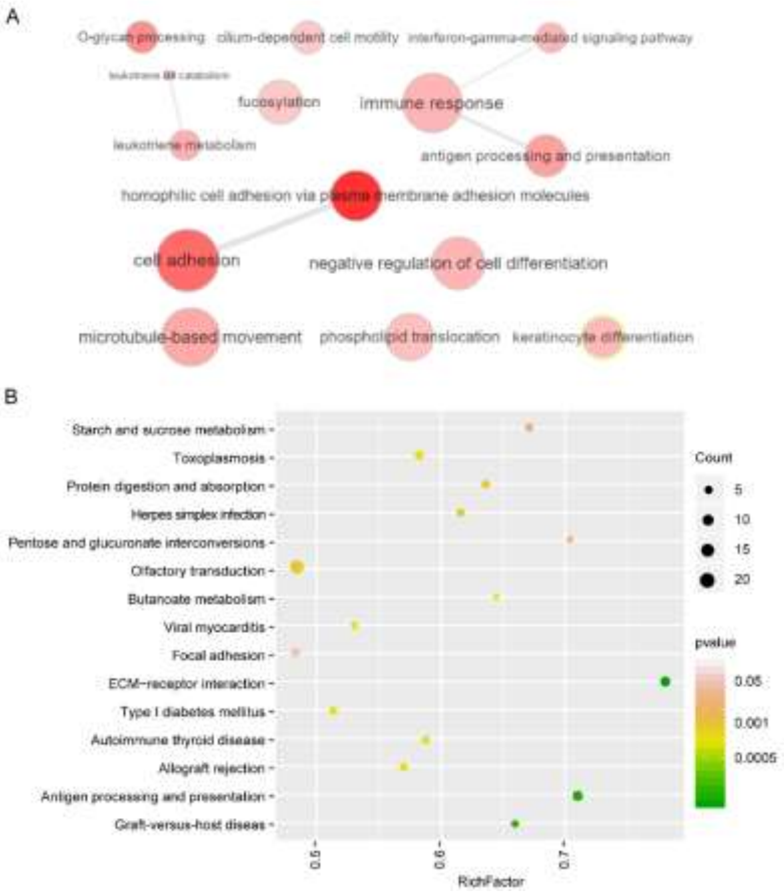

Supplementary Figure S2. Gene Ontology analysis of the somatic mutation genes in Chinese CRC. A, Biological process analysis of the top 500 mutated genes with DAVID 6.8. GO terms were visualized with REVIGO. B, KEGG pathway analysis of the top 500 mutated genes.

Supplementary Table S1. Clinical information of 63 CRC samples

Case No.	Gender	Histology	Metastatic location
C-10	Male	Adenocarcinoma	Liver, Lungs, Lymph nodes
C-103	Male	Adenocarcinoma	Null
C-104	Male	Adenocarcinoma	Lymph nodes
C-105	Male	Adenocarcinoma	Yes
C-107	Male	Adenocarcinoma	Liver
C-109	Male	Adenocarcinoma	Liver
C-11	Male	Adenocarcinoma	Lungs
C-111	Female	Adenocarcinoma	Null
C-112	Male	Adenocarcinoma	Lungs
C-113	Male	Adenocarcinoma	Lungs
C-114	Male	Adenocarcinoma	Liver, Lungs
C-116	Male	Adenocarcinoma	Lungs
C-121	Male	Adenocarcinoma	Lymph nodes
C-123	Male	Adenocarcinoma	Liver
C-126	Male	Adenocarcinoma	Liver
C-127	Male	Adenocarcinoma	Liver
C-128	Female	Adenocarcinoma	Null
C-129	Female	Adenocarcinoma	Lymph nodes
C-130	Male	Adenocarcinoma	Lymph nodes
C-134	Male	Adenocarcinoma	Lungs
C-136	Female	Adenocarcinoma	Lungs
C-137	Female	Adenocarcinoma	Liver
C-138	Female	Adenocarcinoma	Liver
C-139	Female	Adenocarcinoma	Yes
C-140	Female	Adenocarcinoma	Null
C-141	Male	Adenocarcinoma	Liver, Lungs
C-144	Female	Adenocarcinoma	Liver, Lungs

C-145	Male	Adenocarcinoma	Liver
C-146	Male	Adenocarcinoma	Liver
C-147	Female	Adenocarcinoma	Liver
C-148	Male	Adenocarcinoma	Null
C-149	Female	Adenocarcinoma	Liver
C-15	Female	Adenocarcinoma	Lymph nodes
C-151	Male	Adenocarcinoma	Liver
C-2	Male	Adenocarcinoma	Liver
C-20	Female	Adenocarcinoma	Lymph nodes
C-22	Female	Adenocarcinoma	Lymph nodes
C-25	Female	Adenocarcinoma	Lymph nodes
C-26	Female	Adenocarcinoma	Liver, Lungs
C-3	Male	Adenocarcinoma	Liver, Lungs, Bones
C-44	Male	Adenocarcinoma	Lymph nodes
C-46	Male	Adenocarcinoma	Liver
C-49	Male	Adenocarcinoma	Lymph nodes
C-69	Female	Adenocarcinoma	Null
C-70	Female	Adenocarcinoma	Lungs
C-72	Female	Adenocarcinoma	Liver
C-74	Male	Adenocarcinoma	Null
C-75	Female	Adenocarcinoma	Lungs
C-78	Male	Adenocarcinoma	Liver, Lymph nodes
C-81	Female	Adenocarcinoma	Yes
C-82	Male	Adenocarcinoma	Liver
C-84	Male	Adenocarcinoma	Lymph nodes
C-85	Male	Adenocarcinoma	Lymph nodes
C-87	Male	Adenocarcinoma	Null
C-88	Male	Adenocarcinoma	Lymph nodes
C-89	Male	Adenocarcinoma	Null

C-9	Female	Adenocarcinoma	Lungs
C-90	Male	Adenocarcinoma	Liver
C-91	Female	Adenocarcinoma	Lymph nodes
C-92	Male	Adenocarcinoma	Liver
C-94	Female	Adenocarcinoma	Lymph nodes
C-96	Male	Adenocarcinoma	Lymph nodes
C-97	Male	Adenocarcinoma	Null

Supplementary Table S2. Coverage and depth of sequencing

Sample	Raw Reads	Clean Reads	Raw Bases(G)	Clean Bases(G)	Effective Rate(%)	N(ppm)	Q20(%)	Q30(%)	GC(%)	Mean depth(X)	Mean coverage(%)
C-10	126397537	118419215	18.5	17.06	92.22	81.67	98.55	95.42	49.12	289	95.92
C-103	69517312	67067775	10.13	9.68	95.56	173.05	98.86	96.41	47.22	154	95.73
C-104	91236678	87914649	13.43	12.82	95.46	168.73	98.88	96.43	47.78	214	94.48
C-105	93097546	85375493	13.7	12.35	90.15	85.47	98.47	95.13	47.25	200	95.94
C-107	92028692	87622333	13.62	12.81	94.05	165.91	98.75	96.04	50.55	215	94.6
C-109	110759577	102993724	16.33	14.94	91.49	87.86	98.62	95.47	49.12	248	95.96
C-11	91740949	83402887	13.58	12.12	89.25	76.22	98.41	95.03	52.12	202	94.73
C-111	107444439	100463126	15.62	14.39	92.13	79.77	98.64	95.5	47.86	245	95.77
C-112	93061212	85155136	13.72	12.34	89.94	84.4	98.48	95.13	47.5	200	95.87
C-113	71137642	67280340	10.35	9.67	93.43	168	98.75	96.1	49.08	145	94.22
C-114	82016782	78483478	12	11.36	94.67	170.88	98.84	96.37	48.15	176	95.71
C-116	70763282	68105058	10.33	9.85	95.35	169.47	98.85	96.35	48.16	155	95.68
C-121	85317968	78509315	12.59	11.39	90.47	88.22	98.57	95.4	46.17	167	95.76
C-123	77527611	69830056	11.47	10.13	88.32	84.86	98.41	94.94	46.42	160	94.21
C-126	74781900	69459228	11.07	10.12	91.42	87.31	98.55	95.31	46.77	162	95.86
C-127	67533779	63634604	9.88	9.19	93.02	165.78	98.75	96.1	48.58	128	94.15
C-128	82111137	77077250	12.19	11.28	92.53	89.36	98.66	95.6	47.19	188	95.77
C-129	111762514	104873386	16.66	15.4	92.44	89.87	98.7	95.73	48.39	265	95.96
C-130	79990470	72374409	11.89	10.56	88.81	84.8	98.47	95.14	46.76	157	94.36
C-134	59559845	56486722	8.69	8.15	93.79	166.38	98.74	96.03	49.63	139	94.49
C-136	69966929	67753517	10.18	9.76	95.87	174.38	98.88	96.5	46.84	156	94.3
C-137	96823718	90484912	14.24	13.11	92.06	89.44	98.64	95.56	47.69	226	94.17
C-138	70545545	67791865	10.19	9.7	95.19	172.81	98.88	96.47	48.33	168	95.79
C-139	100073189	93115345	14.92	13.68	91.69	87.2	98.6	95.41	46.75	228	94.2
C-140	70869844	68100261	10.14	9.64	95.07	177.33	98.92	96.59	48.53	152	95.46
C-141	79975894	74933697	11.91	11	92.36	90.55	98.71	95.75	47.88	182	94.38

C-144	64573965	61821451	9.26	8.77	94.71	171.8	98.83	96.34	48.7	139	95.49
C-145	79482418	76556433	11.57	11.03	95.33	171.51	98.83	96.31	49.16	189	95.71
C-146	78354740	75713603	11.37	10.88	95.69	173.68	98.86	96.41	47.86	176	94.29
C-147	72007544	69073609	10.7	10.14	94.77	165.63	98.75	96.08	49.29	172	95.78
C-148	56708023	53118621	8.16	7.55	92.52	168.78	98.7	95.98	49.26	108	94.09
C-149	88804535	85584773	12.96	12.36	95.37	172.56	98.86	96.4	48.2	208	95.77
C-15	85435671	77567276	11.73	10.5	89.51	91	98.48	95.09	53.05	172	95.46
C-151	69790050	67132647	10.14	9.65	95.17	172.51	98.84	96.37	48.13	165	95.78
C-2	101583999	94408030	14.87	13.59	91.39	77.95	98.51	95.34	48.78	217	96.28
C-20	105087277	98235845	14.55	13.39	92.03	87.53	98.72	95.77	51.3	174	95.44
C-22	58368884	53423960	8.19	7.48	91.33	94.55	98.66	95.58	51.04	109	93.85
C-25	93868015	86672980	13.68	12.41	90.72	79.13	98.56	95.25	49.39	207	95.72
C-26	84546319	78941728	11.99	11.03	91.99	92.78	98.66	95.63	50.17	165	95.43
C-3	163689279	151647693	24.38	22.2	91.06	74.55	98.46	95.15	51.51	304	94.85
C-44	82389194	75965837	12.05	10.91	90.54	77.08	98.47	95.2	50.47	170	96.15
C-46	131859462	124638696	19.2	17.89	93.18	82.61	98.66	95.69	51.25	289	95.83
C-49	97517247	88534854	14.19	12.64	89.08	77.13	98.45	94.95	49.2	206	95.74
C-69	55245337	50423751	8.22	7.36	89.54	76.39	98.45	94.97	47.84	110	95.82
C-70	94051359	88201528	13.75	12.7	92.36	84.06	98.59	95.58	47.35	199	95.87
C-72	76565020	72915320	11.23	10.57	94.12	167.54	98.8	96.25	48	163	94.23
C-74	70270311	67235463	10.15	9.61	94.68	174.16	98.87	96.47	48.07	140	95.7
C-75	68255674	65545296	10	9.5	95	171.03	98.86	96.39	48.67	164	95.8
C-78	66745300	64219473	9.72	9.25	95.16	172.59	98.84	96.37	46.77	155	95.74
C-81	72252660	69468010	10.56	10.06	95.27	172.89	98.89	96.47	48	169	95.8
C-82	69236210	63546466	10.33	9.32	90.22	72.45	98.37	94.79	46.71	144	95.84
C-84	67149557	63238321	9.54	9	94.34	176.81	98.9	96.55	47.48	124	95.6
C-85	101375597	94755464	15.14	13.93	92.01	79.09	98.61	95.38	47.67	229	95.83
C-87	82710653	76349307	12.13	11	90.68	79.03	98.56	95.28	47.3	177	95.93
C-88	78067018	74669518	11.33	10.73	94.7	173.98	98.88	96.47	48.01	183	95.83

C-89	67054934	64020028	9.72	9.17	94.34	172.84	98.83	96.3	49.89	156	95.93
C-9	94583795	88033729	13.92	12.75	91.59	81.93	98.5	95.31	48.75	210	95.96
C-90	73277295	70399565	10.65	10.11	94.93	172.76	98.82	96.31	48.31	173	95.91
C-91	76588678	73187183	11.22	10.62	94.65	172.46	98.87	96.42	47.61	175	94.41
C-92	85108188	82282593	12.31	11.78	95.69	176.07	98.89	96.52	47.19	190	95.72
C-94	134796523	124760753	20.14	18.32	90.96	76.47	98.54	95.2	47.03	302	95.96
C-96	99877438	96111376	14.6	13.9	95.21	170.5	98.87	96.39	48.66	241	95.8
C-97	87183695	83556298	12.89	12.22	94.8	167.59	98.81	96.25	47.45	200	96.07

Supplementary Table S3. Classifications of variants

**Tumor Sample**	**Nonframeshift deletion**	**Nonframeshift insertion**	**Frameshift deletion**	**Frameshift insertion**	**InDel**	**Specify InDels/Mb**	**Nonsynonymous SNV**	**Synonymous SNV**	**Stopgain**	**Splicing**	**SNV**	Specify SNVs/Mb	Total	Total_mutations/Mb
C-109	29	22	30	21	102	2.9800238	2974	2894	39	17	5924	173.07511	6026	176.05513
C-94	32	15	37	25	109	3.1843183	2621	2443	36	21	5121	149.60453	5230	152.78885
C-139	37	13	267	78	395	11.564808	2574	2079	86	25	4764	139.48037	5159	151.04518
C-129	30	26	24	13	93	2.7170039	2461	2273	36	14	4784	139.76502	4877	142.48202
C-82	25	19	25	15	84	2.4553571	2292	2133	28	21	4474	130.77699	4558	133.23235
C-137	38	15	216	65	334	9.7843268	2157	1671	57	26	3911	114.57037	4245	124.35469
C-128	20	15	72	24	131	3.8306006	1774	1600	31	14	3419	99.97575	3550	103.80635
C-111	15	12	11	15	53	1.5506019	1658	1532	28	10	3228	94.440431	3281	95.991033
C-104	25	11	206	60	302	8.8393172	1573	1112	57	21	2763	80.870971	3065	89.710288
C-141	19	16	22	9	66	1.9306639	1511	1417	28	10	2966	86.762868	3032	88.693532
C-3	37	16	35	24	112	3.2673977	1471	1375	21	12	2879	83.989626	2991	87.257023
C-105	22	14	17	21	74	2.1625545	1502	1356	26	10	2894	84.573415	2968	86.735969
C-85	20	16	21	15	72	2.1057693	1493	1345	22	11	2871	83.967553	2943	86.073322
C-87	22	17	13	16	68	1.987325	1431	1383	27	8	2849	83.263071	2917	85.250396
C-121	17	15	14	12	58	1.6961868	1461	1331	17	8	2817	82.382041	2875	84.078227
C-25	29	10	23	11	73	2.1359968	1329	1377	20	9	2735	80.02673	2808	82.162727
C-70	15	10	18	11	54	1.5776871	1399	1239	21	14	2673	78.09551	2727	79.673197
C-112	20	13	15	11	59	1.7247467	1271	1225	19	9	2524	73.784081	2583	75.508827
C-15	20	16	12	12	60	1.7579728	1169	1228	17	10	2424	71.022103	2484	72.780076
C-130	21	17	19	23	80	2.3403168	1214	1124	20	7	2365	69.185614	2445	71.525931
C-123	20	15	9	12	56	1.6400432	1192	1137	15	12	2356	68.99896	2412	70.639004
C-10	23	11	18	25	77	2.2505564	1101	1047	14	4	2166	63.30786	2243	65.558417
C-49	19	8	12	7	46	1.34545	1051	1018	7	13	2089	61.100981	2135	62.446431
C-20	15	11	21	30	77	2.2560794	975	1017	12	14	2018	59.126861	2095	61.38294
C-69	13	11	9	5	38	1.1095348	1024	950	12	14	2000	58.396566	2038	59.506101

C-2	23	8	22	16	69	2.0120616	977	936	17	9	1939	56.541848	2008	58.55391
C-9	16	6	133	63	218	6.3701703	913	706	25	15	1659	48.47758	1877	54.847751
C-146	17	4	95	29	145	4.2479816	860	683	34	9	1586	46.46413	1731	50.712111
C-103	15	9	53	16	93	2.7212801	859	710	22	10	1601	46.846983	1694	49.568263
C-96	16	9	12	16	53	1.5495069	842	738	10	5	1595	46.631387	1648	48.180894
C-107	14	7	14	17	52	1.5204078	760	755	15	4	1534	44.852029	1586	46.372437
C-145	11	14	5	5	35	1.0233976	788	701	14	9	1512	44.210778	1547	45.234176
C-126	10	11	9	2	32	0.9353788	774	727	9	3	1513	44.225878	1545	45.161256
C-97	12	6	7	2	27	0.7882339	769	715	11	3	1498	43.732382	1525	44.520616
C-46	23	10	15	13	61	1.7832162	745	672	9	10	1436	41.978663	1497	43.761879
C-11	14	16	10	7	47	1.3720898	714	693	12	4	1423	41.542208	1470	42.914298
C-26	18	7	13	13	51	1.4949021	722	668	12	7	1409	41.300335	1460	42.795237
C-151	18	6	13	5	42	1.2284747	716	645	11	3	1375	40.217921	1417	41.446396
C-92	11	8	8	7	34	0.9946409	726	634	14	4	1378	40.31221	1412	41.30685
C-74	13	10	10	3	36	1.0534297	738	613	9	7	1367	40.001065	1403	41.054495
C-136	16	4	78	25	123	3.604459	669	507	26	10	1212	35.517108	1335	39.121567
C-149	14	11	9	8	42	1.2284203	656	614	8	8	1286	37.61306	1328	38.84148
C-91	15	11	10	11	47	1.3755295	660	563	11	7	1241	36.319831	1288	37.695361
C-44	21	7	5	4	37	1.0799672	640	595	5	4	1244	36.310247	1281	37.390214
C-138	15	7	6	4	32	0.9359138	636	579	14	9	1238	36.208164	1270	37.144078
C-114	11	5	11	8	35	1.0241846	635	569	11	4	1219	35.670887	1254	36.695072
C-140	12	5	6	3	26	0.7619014	593	565	12	1	1171	34.314867	1197	35.076769
C-72	11	15	4	7	37	1.0836139	557	554	9	7	1127	33.006293	1164	34.089907
C-88	12	8	8	8	36	1.0526249	573	537	8	5	1123	32.836049	1159	33.888674
C-116	11	6	7	3	27	0.7902222	587	525	9	8	1129	33.042996	1156	33.833218
C-81	12	8	10	11	41	1.1986108	542	486	14	4	1046	30.579192	1087	31.777803
C-134	15	8	9	1	33	0.9658138	511	514	11	4	1040	30.43777	1073	31.403584
C-84	13	5	6	2	26	0.7612669	547	474	10	5	1036	30.333557	1062	31.094824
C-144	13	5	6	5	29	0.8498409	547	467	10	6	1030	30.184005	1059	31.033846

C-89	19	2	7	8	36	1.05216	535	459	10	5	1009	29.489707	1045	30.541867
C-90	13	7	9	7	36	1.0521109	528	469	9	2	1008	29.459105	1044	30.511216
C-147	11	3	3	9	26	0.7605005	514	450	7	5	976	28.548018	1002	29.308518
C-22	13	7	12	11	43	1.2612415	488	390	17	6	901	26.427409	944	27.688651
C-127	15	11	9	4	39	1.142901	474	416	7	4	901	26.403944	940	27.546845
C-75	9	5	14	4	32	0.9359587	423	397	1	6	827	24.188683	859	25.124642
C-113	6	5	8	0	19	0.5563842	452	367	12	1	832	24.363771	851	24.920155
C-78	6	5	5	2	18	0.526642	311	288	8	4	611	17.876571	629	18.403213
C-148	9	9	8	5	31	0.9087928	314	273	6	2	595	17.442958	626	18.351751

Supplementary Table S4. List of 16 somatic mutation genes

Gene	Chinese CRC	TCGA	SOSMIC
HYDIN	88.89%	18.16%	6.55%
FLG	88.89%	23.41%	14.29%
FCGBP	87.30%	14.04%	NA
MUC16	85.71%	35.58%	22.83%
OBSCN	85.71%	26.59%	16.39%
PDE4DIP	82.54%	7.68%	5.23%
TNXB	73.02%	13.86%	8.97%
MUC12	73.02%	0.19%	1.81%
MUC5B	71.43%	18.91%	9.87%
ADAMTS7	71.43%	5.99%	3.86%
TTN	69.84%	63.86%	39.57%
NBPF1	66.67%	2.06%	NA
TP53	36.50%	64.42%	43.33%
APC	31.70%	79.96%	44.53%
KRAS	15.87%	42.88%	33.05%
PIK3CA	12.70%	27.34%	13.65%

Table S5. Significantly mutated genes in Chinese CRC

Gene	Expression	Replication time	hic	n_nonsilent	n_silent	n_noncoding	nnei	p	q	Sample Percent(%)
**NBPF1**	675669	259	40	71	26	34	5	2.10486E-09	6.17283E-07	66.67
**CYP2A7**	1187304	275	31	98	8	22	2	1.40146E-10	6.29389E-08	61.9
**PSG9**	312803	148	-41	51	18	10	0	1.08643E-06	0.000147426	61.9
**KRTAP1-5**	977588	404	10	59	6	1	2	1.77636E-15	1.97092E-12	60.32
**KRT10**	836683	269	-10	62	0	0	1	2.55351E-15	2.53497E-12	55.56
**KCNG4**	582429	364	12	42	3	0	3	6.66134E-16	1.04705E-12	52.38
**ZNF808**	546511	1005	-53	44	0	0	0	9.18573E-07	0.000126988	52.38
MAGEC1	17504	NA	-11	48	2	0	0	1.2952E-06	0.000174501	52.38
LILRB4	978578	625	-1	34	3	1	0	1.19056E-08	2.7724E-06	49.21
NEFH	723458	203	35	33	5	0	3	2.6017E-12	1.63577E-09	47.62
UGT2B11	18988	929	1	45	22	9	0	1.57783E-09	4.80018E-07	47.62
PSG11	263820	178	-32	53	8	18	0	9.14502E-12	5.07333E-09	46.03
OR4A15	1067	1102	-42	30	0	0	1	1.6694E-11	8.74672E-09	46.03
CYP4A11	492091	578	5	31	3	12	5	4.83437E-10	1.72049E-07	46.03
TRDN	23697	1090	-16	34	0	19	1	3.56969E-06	0.000410314	46.03
ACSM2A	181483	823	6	32	0	21	3	1E-18	1E-16	42.86
AGAP5	NA	NA	NA	38	0	6	2	1.68363E-09	5.04073E-07	42.86
TTLL6	727820	415	24	42	2	0	0	1.82551E-07	3.12217E-05	42.86
GSPT1	564730	294	51	29	1	0	0	4.33757E-07	6.70617E-05	42.86
SIGLEC12	360870	583	-4	34	2	4	3	5.26218E-07	7.8774E-05	42.86
TPTE	30271	1268	-81	51	17	95	0	3.96259E-06	0.000442263	42.86
AKNA	218424	360	14	42	6	10	4	7.91352E-06	0.000806836	42.86
ACADS	1741363	349	42	27	1	12	2	1.11022E-16	1.90373E-13	41.27
PRG4	265627	419	-24	39	3	6	4	1.67405E-10	7.34323E-08	41.27
UNC93A	254009	704	1	36	3	6	0	1.83735E-07	3.12217E-05	41.27
RAET1L	402308	382	51	32	4	0	2	2.22064E-09	6.25159E-07	39.68
RBM19	198554	673	11	47	9	14	0	4.50803E-06	0.000500179	39.68

ZNF225	554986	634	-28	26	0	0	1	4.95399E-06	0.000540128	39.68
PRAMEF1	141239	571	18	28	10	7	0	7.51756E-06	0.000770632	39.68
OR2T3	112460	903	-20	25	0	3	5	1E-18	1E-16	38.1
OR9G1	NA	NA	NA	31	0	0	6	1.46549E-14	1.31629E-11	38.1
TUBB1	978901	125	28	37	0	0	2	4.17205E-11	2.01778E-08	38.1
IBSP	423426	538	1	35	1	0	2	2.15993E-09	6.17283E-07	38.1
MSH3	319054	752	36	33	0	46	0	4.716E-07	7.17365E-05	38.1
ZNF302	910826	607	-54	42	0	10	2	8.76856E-07	0.000122513	38.1
LCE1F	64902	818	18	24	0	0	1	1E-18	1E-16	36.51
**TP53**	2069567	213	34	32	0	2	3	1E-18	1E-16	36.51
KIAA1586	431412	348	29	37	0	1	1	5.52095E-09	1.42652E-06	36.51
KRT35	1445853	325	-22	26	2	1	5	1.95819E-07	3.26862E-05	36.51
MTX2	239999	888	11	25	0	10	1	6.22492E-06	0.000658888	36.51
TRIM64B	NA	NA	NA	22	0	0	6	1.33227E-15	1.67528E-12	34.92
NFKBIE	1037256	312	54	35	0	0	0	4.77472E-08	9.58093E-06	34.92
THSD7B	11902	824	-16	25	15	24	1	2.76414E-06	0.000329983	34.92
DNM1L	307288	308	-5	21	20	5	2	2.44249E-15	2.53497E-12	33.33
IGLL1	604420	399	31	22	0	0	17	2.59792E-14	2.22736E-11	33.33
CBWD6	NA	NA	NA	22	1	1	1	1.34617E-06	0.000178813	33.33
SERPINE1	1066804	272	39	22	0	0	0	1.47371E-06	0.000194386	33.33
BPI	562486	350	45	26	8	4	2	2.1729E-06	0.00026964	33.33
STK33	314196	551	13	29	3	0	0	3.14677E-06	0.000365818	33.33
GRIK1	71816	904	-14	22	2	15	0	4.65238E-06	0.000510192	33.33
MARK2	1321109	201	51	23	4	3	8	8.65572E-06	0.00087307	33.33
SLC27A3	1687837	175	33	20	0	2	9	1.22125E-15	1.64537E-12	31.75
**APC**	247811	399	28	24	0	6	4	5.55778E-13	4.19323E-10	31.75
NIPSNAP3B	194451	718	-21	30	0	3	5	1.65934E-12	1.1178E-09	31.75
H3F3C	271605	394	20	24	0	2	5	2.92408E-10	1.12559E-07	31.75
ZNF679	NA	NA	NA	30	3	1	2	2.95492E-08	6.3336E-06	31.75

RYBP	102513	607	32	19	0	6	2	2.9976E-15	2.82704E-12	30.16
WBP1	1069803	343	45	20	0	8	0	1.26232E-13	1.03522E-10	30.16
NBPF11	NA	NA	NA	20	0	0	0	1.37723E-12	9.62124E-10	30.16
MTHFSD	70370	416	3	31	1	1	10	2.29494E-12	1.49266E-09	30.16
DDX27	886628	261	50	21	1	5	0	2.68481E-10	1.07747E-07	30.16
C10orf25	75634	905	25	19	0	0	19	4.71992E-10	1.71206E-07	30.16
OR2J3	155147	738	1	26	1	0	11	2.89531E-09	7.80161E-07	30.16
SLC22A14	301168	437	22	28	0	4	2	1.10797E-07	2.00947E-05	30.16
RHBG	2199280	201	27	19	2	0	3	7.92805E-07	0.000111596	30.16
NPBWR1	99395	697	-19	23	0	0	11	1.79308E-11	9.14082E-09	28.57
KRT4	1781225	288	27	18	0	0	1	1.04343E-09	3.33581E-07	28.57
FSCN3	411207	619	-7	22	0	1	4	1.67522E-08	3.7174E-06	28.57
SASH1	77939	454	-8	18	17	5	1	4.87647E-08	9.6821E-06	28.57
FAM151A	723136	415	20	22	3	0	5	1.13561E-07	2.03999E-05	28.57
RTKN2	111669	382	9	18	0	0	2	1.5055E-07	2.67894E-05	28.57
NAPB	188695	688	-19	18	0	0	1	6.16288E-07	9.08159E-05	28.57
DENND4C	499163	277	13	24	0	9	0	9.76624E-07	0.000133486	28.57
MYOF	458453	380	16	25	6	15	0	1.73121E-06	0.0002252	28.57
KYNU	48515	369	20	21	0	4	2	2.09597E-06	0.000261816	28.57
AMACR	270768	793	4	19	0	2	7	2.51654E-06	0.000302337	28.57
RPGRIP1L	248316	406	-15	19	0	1	0	6.21771E-06	0.000658888	28.57
RNASEH2C	4131934	202	53	17	0	1	4	1E-18	1E-16	26.98
NUDT15	324467	567	34	24	0	0	4	8.88178E-16	1.28868E-12	26.98
UGT1A5	NA	NA	NA	31	2	0	1	4.19309E-12	2.55129E-09	26.98
GPSM2	817825	236	19	22	0	15	2	3.88433E-09	1.03192E-06	26.98
PSG5	307971	178	-25	17	1	21	2	7.18088E-09	1.73648E-06	26.98
POTEM	NA	NA	NA	17	1	1	2	1.32625E-08	3.01395E-06	26.98
HAP1	1617880	323	9	24	0	2	3	4.66192E-08	9.45518E-06	26.98
RP1	148553	735	-8	21	1	0	1	6.70683E-08	1.26504E-05	26.98

RFPL1	723458	203	35	18	2	6	1	1.63475E-07	2.88175E-05	26.98
TCF7L2	156660	435	-9	21	2	1	2	9.22352E-07	0.000126988	26.98
SKIV2L	2314693	203	34	25	0	0	0	1.83979E-06	0.000237686	26.98
LIMS1	363356	408	30	17	8	18	1	2.01088E-06	0.000252861	26.98
ARHGAP19	945635	397	26	17	0	2	1	3.16129E-06	0.000365818	26.98
MUC7	264556	861	26	18	0	0	15	1E-18	1E-16	25.4
TXNDC2	431025	168	34	18	0	0	7	7.088E-12	4.17793E-09	25.4
CDR1	18661	NA	-31	16	0	1	7	8.35941E-10	2.71854E-07	25.4
GOLGA6L1	NA	NA	NA	22	0	0	6	6.85725E-09	1.67976E-06	25.4
ARHGAP42	NA	NA	NA	16	0	0	1	7.85147E-09	1.87461E-06	25.4
CASP7	200770	587	0	17	2	0	4	1.76682E-08	3.87508E-06	25.4
VILL	318742	457	24	16	2	12	0	3.23303E-08	6.85185E-06	25.4
OR51A2	65798	1006	-15	24	1	0	8	5.84516E-08	1.12502E-05	25.4
PSMB3	1198833	207	37	17	0	3	3	6.06656E-08	1.15583E-05	25.4
TM4SF20	248187	164	18	16	0	0	2	3.44199E-07	5.50193E-05	25.4
CES1	317734	581	-28	16	0	14	1	4.68615E-07	7.17365E-05	25.4
MANSC1	698798	242	20	19	3	0	3	7.29564E-07	0.00010425	25.4
MYEOV	558031	443	37	27	1	0	5	1.95397E-06	0.000249026	25.4
FER	140251	741	20	16	21	31	0	2.7992E-06	0.000332067	25.4
USP18	521791	289	11	16	0	0	2	5.80085E-06	0.00062168	25.4
TMPRSS7	265735	336	21	20	15	1	3	7.38979E-06	0.000761673	25.4
LPIN3	383021	309	5	18	0	0	3	8.78073E-06	0.000876307	25.4
ARL2	3151458	209	52	15	0	0	15	1.11022E-16	1.90373E-13	23.81
CST4	189028	819	-21	15	2	0	2	1.81563E-10	7.78328E-08	23.81
KRTAP5-2	NA	NA	NA	17	0	1	2	2.04041E-10	8.55248E-08	23.81
CPOX	364371	640	6	19	0	0	7	1.60769E-08	3.61003E-06	23.81
PRKRIR	234429	366	57	19	0	0	7	5.77207E-08	1.1224E-05	23.81
GPR110	203250	291	-5	23	0	0	2	1.02534E-07	1.89607E-05	23.81
KRTAP10-5	867542	461	28	19	0	0	12	1.65515E-07	2.89069E-05	23.81

MMP20	530118	436	17	19	0	0	3	3.98279E-07	6.26029E-05	23.81
PPIG	345417	404	38	15	0	13	4	1.31235E-06	0.000175556	23.81
TPRX1	881316	336	39	16	2	0	1	1.97209E-06	0.000249648	23.81
THUMPD1	179233	641	14	16	0	0	2	3.90481E-06	0.000438408	23.81
AKR1C1	343868	608	-13	14	1	6	7	1E-18	1E-16	22.22
ESD	272205	820	63	16	0	1	8	4.12569E-11	2.01778E-08	22.22
TCTEX1D1	335045	692	-16	25	0	3	7	7.94238E-11	3.74523E-08	22.22
PABPN1L	860087	252	37	14	0	2	19	2.87041E-10	1.12559E-07	22.22
SLC38A4	518041	415	-10	14	1	6	3	5.02501E-10	1.75522E-07	22.22
C12orf45	514255	204	37	14	0	0	8	1.75847E-07	3.04297E-05	22.22
FAHD2A	255441	536	19	16	1	1	21	2.11721E-07	3.50306E-05	22.22
CYP20A1	417679	298	23	14	0	0	4	4.08947E-07	6.37484E-05	22.22
DKKL1	2183690	309	30	19	0	0	2	5.93586E-07	8.81593E-05	22.22
POU5F1B	NA	NA	NA	25	0	1	2	6.50582E-07	9.43944E-05	22.22
EHD3	102236	884	15	14	0	2	4	2.46457E-06	0.000299914	22.22
OSCAR	767094	317	-50	14	0	0	2	6.65367E-06	0.000693378	22.22
KRTAP17-1	1149433	468	-14	15	0	0	10	1E-18	1E-16	20.63
SPANXD	NA	NA	NA	24	0	0	6	1.44329E-15	1.70146E-12	20.63
PGF	875817	222	53	13	0	0	3	8.60285E-11	3.95773E-08	20.63
LCE4A	169158	818	24	15	0	0	1	3.63457E-10	1.37111E-07	20.63
ASPHD1	NA	NA	NA	13	3	0	2	3.85254E-10	1.42484E-07	20.63
CCL11	40425	871	-32	13	0	0	3	2.61633E-09	7.25722E-07	20.63
CLIC3	1979517	188	34	14	0	0	5	2.89132E-09	7.80161E-07	20.63
C6orf57	119480	618	15	13	0	0	1	6.23759E-09	1.58991E-06	20.63
C10orf53	NA	NA	NA	13	0	0	1	1.309E-08	3.01102E-06	20.63
FAM91A1	483934	269	10	13	4	1	0	3.96308E-08	8.12518E-06	20.63
N6AMT1	312901	480	6	13	0	2	3	3.09943E-06	0.000363114	20.63
POLN	765279	332	34	13	9	16	3	3.74367E-06	0.00042538	20.63
CA9	1202125	248	37	13	11	0	0	3.79894E-06	0.000429075	20.63

ZDHHC12	1865107	197	38	13	0	0	1	8.75633E-06	0.000876307	20.63
SLFNL1	341851	171	29	17	0	0	3	1.50324E-13	1.18142E-10	19.05
C11orf40	190769	879	-1	12	0	1	1	8.15108E-10	2.69729E-07	19.05
IFT74	116568	447	-12	17	0	0	3	2.14153E-09	6.17283E-07	19.05
LIPF	145689	327	40	12	6	0	6	2.21524E-08	4.80273E-06	19.05
RDM1	703538	424	31	13	0	6	1	3.79092E-08	7.85763E-06	19.05
KRTAP13-2	4608	1035	-69	12	1	0	4	6.50201E-07	9.43944E-05	19.05
CDK5RAP2	386697	450	10	12	5	0	0	6.91063E-07	9.95025E-05	19.05
KRT78	1781225	288	27	19	0	0	1	1.87866E-06	0.000241056	19.05
KRTAP4-5	996799	468	-7	19	1	0	5	6.5539E-06	0.000686776	19.05
WDR63	443738	623	11	17	0	7	1	9.75822E-06	0.000968735	19.05
CWF19L2	147499	853	-10	28	0	11	3	9.06353E-12	5.07333E-09	17.46
C21orf58	940047	624	53	17	0	0	3	2.12752E-10	8.72376E-08	17.46
DMKN	954618	367	35	16	0	4	3	5.27123E-10	1.80775E-07	17.46
PRKCI	361822	270	18	11	2	7	1	9.97406E-08	1.86268E-05	17.46
OXA1L	1153069	351	36	11	9	0	1	2.43028E-07	3.98608E-05	17.46
NENF	416515	283	18	11	0	0	7	4.77176E-07	7.2004E-05	17.46
PBOV1	341233	545	21	12	0	0	35	2.4808E-06	0.000299954	17.46
**KRAS**	259193	512	16	11	0	0	1	0.003206918	0.1214636	15.87
SEC14L3	657636	288	48	12	0	0	4	6.53967E-09	1.63883E-06	15.87
PHC1	296906	416	12	10	1	1	5	6.60328E-09	1.63883E-06	15.87
SLC6A11	225460	545	30	10	6	3	1	5.1222E-06	0.000555258	15.87
CPNE1	1423559	169	53	18	0	1	1	7.11532E-06	0.000737413	15.87
PPAP2C	233160	578	-13	9	0	0	14	7.96141E-13	5.7757E-10	14.29
H2BFM	NA	NA	NA	9	0	0	50	1.11259E-11	5.9959E-09	14.29
NACA2	323585	358	-8	25	1	0	8	6.64195E-10	2.23715E-07	14.29
FBXL8	1093307	150	47	9	0	1	5	5.38293E-09	1.41018E-06	14.29
MYCT1	188249	600	-9	9	0	0	2	3.36635E-08	7.05512E-06	14.29
CD68	1957426	194	24	9	0	8	2	2.69544E-07	4.38288E-05	14.29

PLA2G2C	608368	501	27	8	0	0	1	1.56224E-09	4.80018E-07	12.7
CYP3A5	1395063	430	34	8	5	2	5	5.23316E-08	1.02821E-05	12.7
CYB561	874574	267	-20	8	0	1	4	1.0483E-07	1.91972E-05	12.7
WDR70	302307	592	15	8	0	2	1	1.92174E-07	3.23641E-05	12.7
DLX2	534370	295	34	9	0	1	3	2.98768E-06	0.00035221	12.7
TMEM87B	564343	466	36	8	0	9	4	8.41021E-06	0.000852867	12.7
C16orf52	NA	NA	NA	7	0	0	50	1E-18	1E-16	11.11
C19orf25	2352825	221	38	7	0	0	21	1.55088E-09	4.80018E-07	11.11
SPG21	705779	185	57	7	0	0	4	9.45503E-09	2.22926E-06	11.11
PRTFDC1	164255	142	18	7	0	2	3	7.55609E-07	0.00010716	11.11
CD3G	963668	192	48	7	1	0	6	1.63174E-06	0.000213735	11.11
CD300LG	1025836	241	19	7	0	0	1	2.19203E-06	0.000270236	11.11
CLEC18C	1353954	139	37	7	1	1	3	2.3978E-06	0.000293683	11.11
CCL26	1012112	255	51	7	0	1	3	3.72404E-07	5.90277E-05	9.52
GCH1	673378	380	40	6	0	2	12	3.58932E-06	0.000410314	9.52
C10orf88	282374	619	13	6	0	0	7	6.25284E-06	0.000658888	9.52
C14orf178	429207	388	37	5	0	0	7	3.43714E-07	5.50193E-05	7.94
NKX6-2	278505	581	-12	5	0	0	18	4.61568E-06	0.000509128	7.94
MGST2	382197	256	12	10	0	19	0	5.69371E-06	0.000613685	6.35

Table S6. Driver genes analyzed by OncodriveFM and MutSigCV

Unnamed: 0	PVALUE	QVALUE	SIFT_PVALUE	PPH2_PVALUE	MA_PVALUE	Hugo_Symbol
ENSG00000013016	6.16E-07	6.74E-06	0.0015	0.0016	0.0012	EHD3
ENSG00000100285	0	0	1.00E-08	1.00E-08	1.00E-08	NEFH
ENSG00000101162	0	0	1.00E-08	1.00E-08	1.00E-08	TUBB1
ENSG00000103248	1.91E-07	2.18E-06	0.0008	0.0011	0.0009	MTHFSD
ENSG00000103494	0.000191	0.001316	0.0115	0.0137	0.012	RPGRIP1L
ENSG00000107159	0.00049	0.002848	0.0198	0.0158	0.0182	CA9
ENSG00000111961	0.00049	0.002848	0.0198	0.0158	0.0182	SASH1
ENSG00000116690	4.71E-05	0.000346	0.0079	0.0068	0.007	PRG4
ENSG00000117691	7.16E-14	1.19E-12	0.0001	1.00E-08	0.0001	NENF
ENSG00000119630	0	0	1.00E-08	1.00E-08	1.00E-08	PGF
ENSG00000128322	0.00018	0.001244	0.0104	0.0134	0.0127	IGLL1
ENSG00000132677	0	0	1.00E-08	1.00E-08	1.00E-08	RHBG
ENSG00000137674	7.79E-09	9.60E-08	0.0004	0.0003	0.0002	MMP20
ENSG00000141510	0	0	1.00E-08	1.00E-08	1.00E-08	TP53
ENSG00000151131	0	0	1.00E-08	1.00E-08	1.00E-08	C12orf45
ENSG00000160298	0.000276	0.001811	0.0131	0.0157	0.0141	C21orf58
ENSG00000160654	1.38E-13	2.23E-12	0.0001	1.00E-08	0.0002	CD3G
ENSG00000165511	0	0	1.00E-08	1.00E-08	1.00E-08	C10orf25
ENSG00000168418	5.12E-13	7.84E-12	1.00E-08	0.0002	0.0004	KCNG4
ENSG00000168955	0.000384	0.002466	0.0171	0.0167	0.015	TM4SF20
ENSG00000170423	0.001245	0.006892	0.0265	0.0266	0.0245	KRT78
ENSG00000170909	3.38E-07	3.80E-06	0.0015	0.0011	0.0009	OSCAR
ENSG00000171790	0.00047	0.002848	0.0157	0.0189	0.0183	SLFNL1
ENSG00000172927	0	0	1.00E-08	1.00E-08	1.00E-08	MYEOV

ENSG00000173805	1.10E-06	1.14E-05	0.002	0.0013	0.0021	HAP1
ENSG00000181958	0	0	1.00E-08	1.00E-08	1.00E-08	OR4A15
ENSG00000182333	0	0	1.00E-08	0.0002	1.00E-08	LIPF
ENSG00000183729	0	0	1.00E-08	1.00E-08	1.00E-08	NPBWR1
ENSG00000186860	0.00049	0.002848	0.0198	0.0158	0.0182	KRTAP17-1
ENSG00000188375	0	0	1.00E-08	1.00E-08	1.00E-08	H3F3C
ENSG00000196539	0.001732	0.009178	0.0313	0.0289	0.0284	OR2T3
ENSG00000197079	6.05E-08	7.22E-07	0.0007	0.0008	0.0004	KRT35
ENSG00000198848	0	0	1.00E-08	0.0002	1.00E-08	CES1
ENSG00000204790	0	0	1.00E-08	1.00E-08	1.00E-08	CBWD6
ENSG00000205022	6.53E-07	6.95E-06	0.0016	0.0012	0.0016	PABPN1L
ENSG00000205496	0	0	0.0003	1.00E-08	1.00E-08	OR51A2
ENSG00000221852	0	0	1.00E-08	1.00E-08	1.00E-08	KRTAP1-5
ENSG00000242110	7.82E-10	1.17E-08	0.0002	0.0001	0.0001	AMACR
ENSG00000254440	0	0	1.00E-08	1.00E-08	1.00E-08	PBOV1
ENSG00000254521	2.33E-06	2.20E-05	0.0018	0.0027	0.0026	SIGLEC12
ENSG00000256294	0	0	1.00E-08	1.00E-08	1.00E-08	ZNF225
ENSG00000263956	0	0	1.00E-08	0.0002	1.00E-08	NBPF11
ENSG00000274391	9.64E-05	0.00068	0.0098	0.0091	0.0096	TPTE
ENSG00000277791	6.92E-07	7.35E-06	0.0021	0.0013	0.0012	PSMB3
